# Reversibility of Hepatic Histological Damage after Surgical Temporary Obstruction of the Common Bile Duct in a Murine Model

**Published:** 2011-03

**Authors:** H. Juárez Olguín, J. L. Figueroa Hernández, D. Calderón Guzman, R. Alemón Medina

**Affiliations:** 1 *Laboratorio de Farmacología, Instituto Nacional de Pediatría, Mexico City, CP 04530, Mexico;*; 2 *Departamento de Farmacología, Facultad de Medicina, Universidad Nacional Autónoma de México. CP 04530, Mexico;*; 3 *Laboratorio de Neuroquímica, Instituto Nacional de Pediatría, Mexico City, CP 04530, Mexico*

**Keywords:** animal model, common bile duct, Balb C mice, reversible hepatic damage

## Abstract

The reversibility of hepatic histological damage after restoring bile flow in a murine model was assessed. 25 male Balb C mice (25-35 g, age 6 weeks) were divided into 5 groups and their common bile duct (CBD) fastened to obstruct the release of gall bladder and liver contents. Group I, CBD untied at day 10, group II at day 15, and groups III and IV at days 20 and 30, respectively. Hematoxilin-eosin stained liver slices were analysed 0, 5, 10 and 20 days after restoring bile flow. Group I showed slight histological lesions (second stage), as cholangiolar bile pigment concretion, pericholangiolar and portal collagen accumulation; group II, mild lesions (third stage), as cholangiolar hamartomatous proliferation and bile duct portal fibrosis; group III showed severe lesions (fourth stage), as loss of functional parenchyma, and also the second and first stage lesions. Group IV died before 30 days. First stage corresponds to absent lesions (control group). Group I recovered totally, group II recovered only from slight lesions and group III had irreversible damage. Severity of lesions increased gradually and accumulatively, irreversible hepatic damage was achieved at 20 days and is deadly at 30 days. Our model of temporary CBD obstruction was suitable to assess reversibility of hepatic histological damage.

## INTRODUCTION

Obstruction of the common bile duct (CBD, ductus choledochus) is known to occur as part of the physiopathology of choledocholithiasis or physical compression by neoplasia or inflammation of nearby organs. This obstruction may disturb the normal hepatic morphology, by increasing the normal pressure inside liver lobules, leading to minor lesions as centrolobular cholestasis, or even more severe damage, as hepatic cirrhosis (1, 2). However, albeit the incidence of CBD obstruction has increased worldwide (3), its involvement in histologic hepatic damage, needs to be revised further, so as to at which extent the possibility of restoring the normal function and structure of the hepatic tissue after removing the obstructing agent can be accomplished. A few studies have been conducted to characterise the evolution of reversibility (recovery) of the affected zones through time (4), and in mice, common bile duct fastening has been thoroughly described as a candidate method to induce ductal hyperplasia (5). Additionally, the results from a previous study, using this model showed that there exists a correlation between the extent and magnitude of the induced disorders and the time the obstruction is present (6).

Severity of lesions increase as the obstruction persists, leading to the appearance of histopathological disorders such as sinusoidal congestion in the early stages and biliary fibrosis with cholangiolar hamartomatous proliferation when obstruction persists even more; these disorders were confirmed with models reported elsewhere (7). This model has been applied to different species, and several advantages have been found after conducting it in mice, such as availability, reproducibility, and well tolerated (8, 9).

Therefore, the aim of this study is to determine the reversibility of hepatic histological damage induced by temporary surgical obstruction of the common bile duct in Balb C mice, as a model of bile flow obstruction in man.

## METHODS

### Animals

25 male Balb C mice, age 6 weeks, weighing 25 to 35 g, bred in laboratory, were used in this study. Animals were divided into 4 groups including 5 animals each, plus a control group with 5 animals having intact CBD, maintained in 36 × 26 × 16 cm acrylic cages under controlled conditions, temperature 21 ± 3°C, 55 ± 10% relative humidity, and 10-12 air changes per hour, with cycles of light/darkness of 12/12 hours and free access to water and food, pine shavings as supporting material. Animals were free of viruses and other pathogens. The research project was approved by the Ethical Committee for the Care of Laboratory Animals (ECCLA) of our Institution.

### Experimental procedure

After being completely anaesthetised with sodium phenobarbital (5.5 mg/kg i.p.), a surgical 1.5 cm length incision was made to all the animals, in the right upper quadrant, getting through the skin, then subcutaneous tissue (ST), superficial and profound aponeurosis, traverse and rectus muscles and peritoneum. A small polyethylenecellulose canule, 3 mm internal diameter and 8 mm length, was inserted into the gallbladder, in order to allow the total recovery of bile flow into the small intestine when untying the CBD. The incision was closed according to two plains, the first with peritoneum, muscles and aponeurotic fascia and, the second, with skin and subcutaneous tissue.

Surgical wounds were cleaned with sterile soap, water and hydrogen peroxide and the animals were put back into their cages directly after the surgery, and heat was given to them with an electric lamp. The first day, bile duct obstruction was carried out to the four experimental groups, except by the control, and the following experimental scheme was conducted: Day 10: the bile duct of group I was untied and liver biopsies were taken. Day 15, the bile duct of group II was untied and liver biopsies from groups I and II were taken. Day 20, group III CBD was untied and liver biopsies were taken from groups I, II and III. Day 30, the common bile ducts from the remaining 5 mice (group IV) were untied and hepatic biopsies were taken from the whole groups.

At the 30^th^ day, biopsies from several organs, such as liver, brain, kidneys and lungs were taken from each mouse, and analysed microscopically. Organs were immersed in formalin (10% v/v methanol) during 48 hrs at least, included in paraffin, sliced into 2 to 8 μm portions, stained with haematoxylin-eosin and photomicrographed. Two pathologists carried out microscopic analyses independently, in a double-blinded manner, and their results were confronted. Severity of the histological damage was categorised into four stages or grades: First, null or absent lesions. Second, slight lesions, including bile pigment concretion and collagen accumulation. Third, mild lesions, including hamartomatous ones. Fourth, severe lesions, including loss of functional parenchyma.

### Statistical analysis

Data corresponding to the stage of histological disturbance among groups were scored in analogous scale with arbitrary units, and numeric values were assigned to each stage. Data were analysed by the ANOVA test, and the significant differences were evaluated by Tukey test (*p*≤0.05).

## RESULTS

Common bile duct surgical obstruction was well tolerated by the mice. The animals from experimental group IV died before the hepatic biopsies could be obtained, probably due to severe hepatic lesions, which lead to liver failure and death.

**Figure 1:**
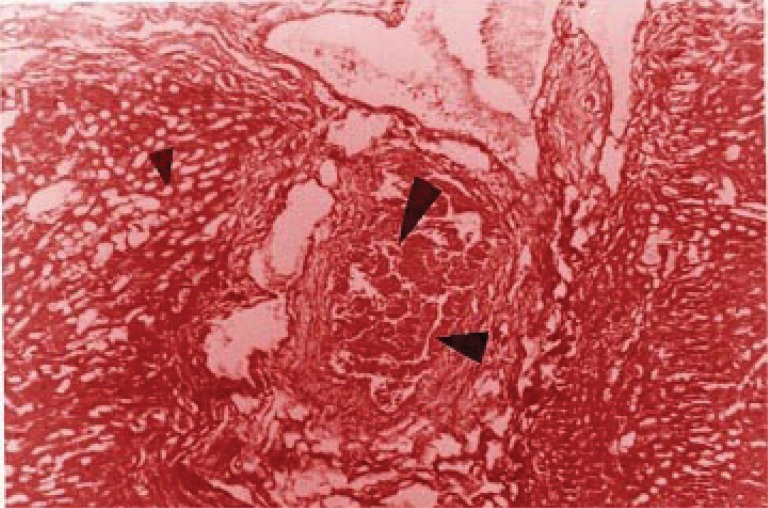
Intracolangiolar biliary thrombus (large arrow) with concentric connective proliferation, epithelial duct fold (medium arrow) and steatosis features (small arrow).

**Figure 2:**
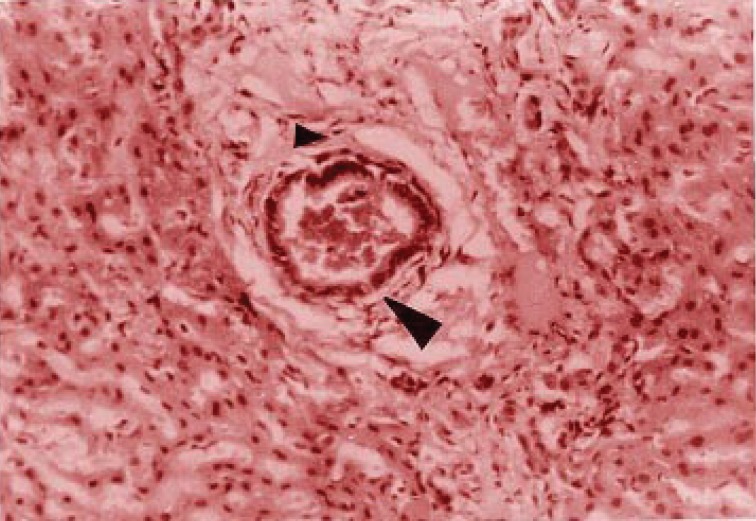
Periductal fibrosis-associated biliary thrombus (large arrow), mononuclear leukocytes and fibroblasts (small arrow).

Slight hepatic lesions, such as cholangiolar bile pigment concretion, as well as pericholangiolar and portal collagen accumulation (Figure 1) were found in 4 of the 5 mice in group I. Group II showed an average of mild (third grade) lesions as cholangiolar hamartomatous proliferation and bile duct portal fibrosis affecting the limiting plaque and lobules (Figure 2); most of the animals in this group showed also second grade lesions, which suggests that these histological disturbances are accumulative. No relevant histological changes were found in the control group, which was then classified as null or absent lesions (first stage).

**Figure 3:**
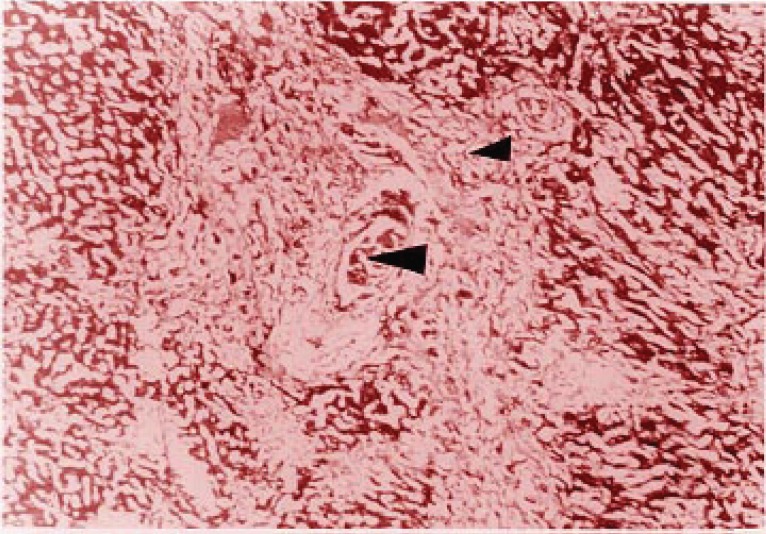
Cholangiolar biliary thrombosis (large arrow) associated to irregular fibrosis augmented in the portal area that involves the lobule (small arrow), mainly centrally located.

Analyses at the 20^th^ day showed a series of disturbances, as described below: At the 10^th^ day, sinusoidal and vascular congestion in the portal area associated to bile hepatocellular stasis were also observed in group I; however, diminished vascular congestion without bile stasis (Figure 3), appearing one week after the untying, suggested damage recovery.

**Figure 4:**
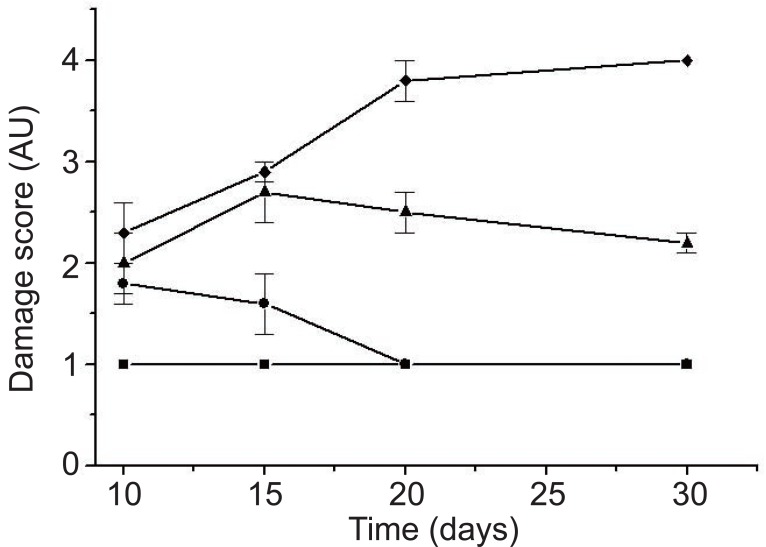
Curse of hepatic lesions with bile flow restoration in Balb C mice. Damage score scale in arbitrary units (AU). (—■—) Control group remained at the first score throughout the study (score 1) with null or absent lesions. (—●—) Group I showed slight lesions (score 2) and showed complete recovery at day 20. (—▲—) Group III showed mild lesions (score 3) and partial recovery to day 30. (—♦—) showed mild to severe damage (score 3 and 4), which remained at that score until day 30 (irreversible damage). Standard deviations are due to the absence of lesions in some individuals within the same group.

**Figure 5:**
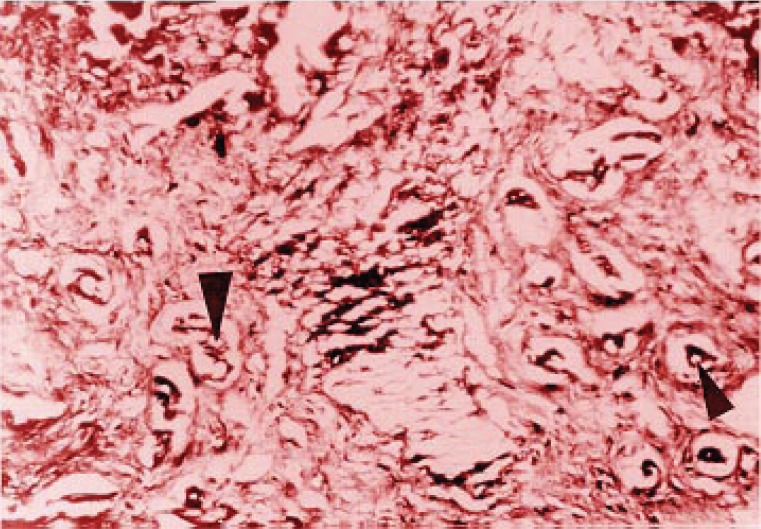
Portally located pseudohamartomatous cholangiolar proliferation (arrow zones).

Lesions observed five days after untying in group II, remained mainly in the third grade, which consisted in cholangiolar hamartomatous proliferation and bile duct portal fibrosis with limiting plaque and lobules deformation (Figure 4). On the other side, functional parenchyma loss and increased bile duct proliferation and fibrosis (Figure 5), were severe lesions, observed in mice of group III, showing that the damage in this group was irreversible.

**Figure 6:**
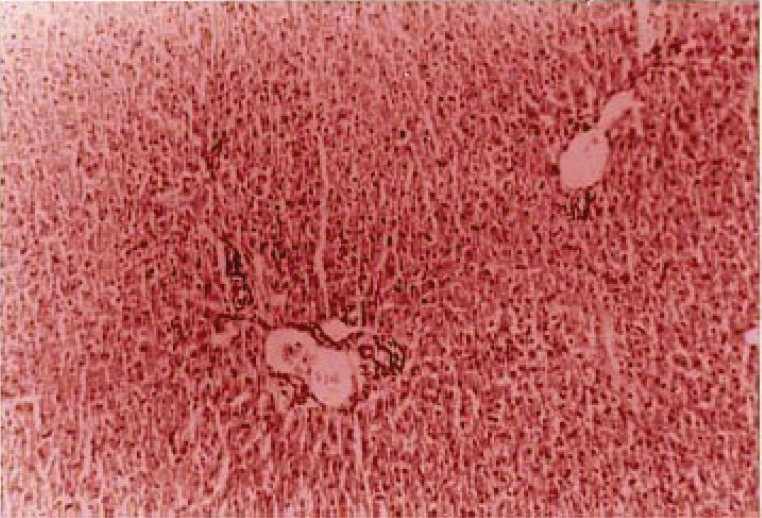
Portal area sinusoidal and vascular congestion, associated to hepatocellular bile stasis in the liver tissue.

Cholangiolar hamartomatous proliferation and limiting plaque and lobules deforming portal fibrosis (Figure 6), were the lesions found in the animals from group II.

Lesions found in the biopsies from the third group resembled those observed in the initial biopsy and remained in that stage until day 30 (Figure 4).

Vascular congestion and dilation of variable magnitude were the only type of damage observed in brain, heart and lung biopsies during the first 15 days; however, bile pigment accumulation was observed within the lumen of collecting tubules, in the kidney biopsies.

## DISCUSSION

In the early stage of this study, when the method to determine the onset and magnitude of hepatic histological disturbances at the expense of common bile duct obstruction was developed, it was established that severe lesions are induced if the obstruction persisted for more than 15 days, and shorter obstruction times induced less damage. In the present work, such preliminary conclusions were ascertained, since the development of mild to severe histological disorders, as those characterised by Wright and Braithwaite (10), and Bioulac et al. (11), could be observed in group II, which occurred at the fifteenth day of obstruction.

Lesions found in group III, untied at day 20, showed severe damage, as compared to groups I and II. As initially stated, our purpose was to investigate the reversibility (recovery) of hepatic histological disturbances after common bile duct obstruction, and 5 days after removing the obstruction (15^th^ day), the entire group I showed an adequate recovery, and the results rendered by both analysts correlated as they observed a noticeable recovery of the lesions 5 days after removing the obstruction. However, such recovery becomes more evident at the 20^th^ day, since the entire group showed no significant histological disturbances. Therefore, special attention was paid to this group, considering the evolution of the lesions found during the 10 day-lasting obstruction and the histological changes that could be observed after bile duct liberation, as seen in previous reports (12, 13).

Helpfulness of the temporary common bile duct obstruction procedure is worth to be highlighted, not just for their feasibility in similar studies, but also in the treatment and outcome evaluations, as has been widely described by other authors, both in murine models (14, 15) or in man (16).

Hepatic histological disturbances such as cholangiolar bile pigment concretion, as well as a slight pericholangiolar and portal collagen accumulation, derived by temporary bile duct obstruction can be reverted. However, further studies are needed to conclude about the total impact of CBD obstruction and hepatic damage.
